# Clinical characteristics of bipolar disorder: a comparative study between Argentina and the United States

**DOI:** 10.1186/s40345-015-0027-z

**Published:** 2015-04-24

**Authors:** Jessica N Holtzman, Maria Lolich, Terence A Ketter, Gustavo H Vázquez

**Affiliations:** Department of Psychiatry and Behavioral Sciences, Stanford University School of Medicine, 401 Quarry Road, Stanford, CA 94305 USA; Department of Neuroscience, Research Center in Neuroscience and Neuropsychology, Universidad de Palermo, Mario Bravo 1259, C1175ABT Buenos Aires, Argentina

**Keywords:** Argentina, Bipolar disorder, Clinical characteristics, Comparative, Early onset, Onset age, Prescription patterns, United States

## Abstract

**Background:**

Bipolar disorder presents with diverse clinical manifestations. Numerous investigators have sought to identify variables that may predict a more severe illness course.

**Methods:**

With the objective of studying the clinical characteristics of bipolar patients between South and North America, a comparison was performed between a sample from Argentina (*n* = 449) and a sample from the United States (*n* = 503) with respect to demographics and clinical characteristics, including presence of comorbidities.

**Results:**

The Argentinian sample had more unfavorable demographics and higher rates of prior psychiatric hospitalization and prior suicide attempt but a better social outcome. However, the sample from the United States had a higher rate of prior year rapid cycling, as well as younger bipolar disorder onset age (mean ± SD, 17.9 ± 8.4 vs. 27.1 ± 11.4 years) and more severe clinical morbidity, though there was no significant difference in terms of the total duration of the illness. Argentinian compared to American patients were taking more mood stabilizers and benzodiazepines/hypnotics, but fewer antipsychotics and other psychotropic medications, when considering patients in aggregate as well as when stratifying by illness subtype (bipolar I versus bipolar II) and by illness onset age (≤21 vs. >21 years). However, there was no significant difference in rate of antidepressant prescription between the two samples considered in aggregate.

**Conclusions:**

Although possessing similar illness durations, these samples presented significant clinical differences and distinctive prescription patterns. Thus, though the Argentinian compared to North American patients had more unfavorable demographics, they presented a better social outcome and, in several substantive ways, more favorable illness characteristics. In both samples, early onset (age ≤ 21 years) was a marker for poor prognosis throughout the illness course, although this phenomenon appeared more robust in North America.

## Background

Bipolar disorder is a severe and enduring condition that affects a significant portion of the population globally (Weissman et al. 1996). Though specific annual rates of occurrence can vary by geographic region of the world (Soutullo et al. 2005), a large, international study of ten countries revealed consistent lifetime rates of bipolar disorder (Weissman et al. 1996). Such studies of the Americas, Europe, and Asia have revealed a 2.4% lifetime prevalence of bipolar spectrum disorders (Merikangas et al. 2011). More specifically, a 2.3% lifetime prevalence of bipolar spectrum disorders was reported in South American countries including Brazil and Colombia, while a 4.4% prevalence was found in the United States (Merikangas et al. 2011). Given the similarities in the lifetime incidence across geographic regions of the world, culture specific risk factors - both social and genetic - may impact the observed cross-regional differences in the comorbidities and clinical expression of bipolar disorder.

Owing to the significant diversity in etiology and clinical presentation of bipolar disorder, investigators have sought to define more homogenous subgroups with the goal of identifying genetic biomarkers, further understanding the neurobiology, and delineating more specifically tailored, and thus more efficacious, treatment regimens (Teixeira et al. 2013; Seifuddin et al. 2012; Geoffroy et al. 2013).

Age at onset of bipolar disorder has been demonstrated as a valid defining characteristic of phenotypically similar subgroups (Strober 1992; Vázquez et al. 2011). A recent Argentinian study found onset age significantly differentiated between unipolar and bipolar affective disorders, consistent with other international studies (Dervic et al. 2014; Tondo et al. 2010). Studies have sought to identify specific characteristics of juvenile-onset bipolar disorder (Geller and Luby 1997). Though early-onset bipolar disorder has been defined variably across studies and remains understudied in terms of specific phenotypic presentation and treatment response, it has been consistently associated with a more severe clinical course and comorbidities (Leverich et al. 2007; Perlis et al. 2004; Leboyer et al. 2005). Thus, childhood-versus-adolescent-and-adult-onset bipolar disorder has been consistently associated with higher rates of comorbid anxiety disorder, prior suicide attempt, substance and alcohol use disorders, rapid cycling, and other validated measures of morbidity (Holtzman et al. 2015; Rende et al. 2007; Goldberg and Ernst 2004).

Despite these consistent findings, investigators have found that apparent rates of diagnosis of childhood onset bipolar disorder may differ between geographic regions of the world, and further that the clinical characteristics of the various childhood onset groups may also differ (Vazquez et al. 2012; Post et al. 2014). Though several potential rationales have been proposed to explain these differences in illness course and comorbidities, including varying levels of psychosocial stress in childhood and differential migration patterns according to genetic vulnerability of affective disorders, no single theory has been shown to possess significantly greater validity than others (Post et al. 2014). Further, patterns of psychopharmacological treatment, including rates of antidepressant and benzodiazepine use, have been demonstrated to differ significantly between European and American samples (Post et al. 2011).

Since the advent of observationally based studies of bipolar disorder clinical course and characteristics, several varying opinions regarding the respective roles of onset age, duration of illness, and number of prior episodes have been proposed. While some studies have proposed illness duration as a direct measure of potential neurodegeneration (Frey et al. 2008), others have supported number of prior episodes as the most accurate predictor of longitudinal outcome (Altman et al. 2006; Goldberg et al. 2005). Still other studies have suggested that onset age is a more robust method of predicting illness course, both due to its demonstrated efficacy in delineating phenotypically distinct subgroups and its putative ability to reflect common genetic characteristics (Alda et al. 2000; Azorin et al. 2013; Baldessarini et al. 2012; Etain et al. 2012).

As such, the main objective of this study was to assess differential clinical characteristics, comorbidities, and treatment patterns between South and North American bipolar disorder patients. Secondarily, the study sought to identify the role of onset age in defining subgroups with a homogenous clinical phenotype, as well as to assess the specific clinical characteristics of these subpopulations. We compared the clinical characteristics of observational, naturalistic, cross-sectional data from South and North American samples in order to assess the consistency in illness profile of early-onset bipolar disorder across geographic regions and to further validate onset age as a potential predictive variable of prognosis in bipolar disorder.

## Methods

The sample of 449 patients from Argentina consisted of patients with bipolar I and II disorder enrolled between 2009 and 2011 in the Argentine Network on Bipolar Disorders, which includes 11 tertiary-care mood disorder clinics throughout Argentina. The sample of 503 patients from the United States similarly consisted of patients with bipolar I and II disorder referred to the Stanford University Bipolar Disorders Clinic between 2000 and 2011. Stanford patients were enrolled in conjunction with the Systematic Treatment Enhancement Program for Bipolar Disorder (STEP-BD) (Sachs et al. 2002; Sachs et al. 2003). All enrolled patients were diagnosed according to the Diagnostic and Statistical Manual of Mental Disorders (DSM)-IV diagnostic criteria and through semi-structured interview (Mini International Neuropsychiatric Interview (MINI) and Structured Clinical Interview for DSM for mood disorders). The protocols for subject enrollment were approved by the local Institutional Review Board (Stanford University Administrative Panel on Human Subjects) of each center. Subjects provided both verbal and written informed consent prior to enrollment and participation.

Assessment of onset age was based on patient retrospective recall of the first occurrence of a syndromal hypomanic, manic, mixed, or major depressive episode. Comparative variables were selected based upon clinical characteristics and comorbidities that were frequently reviewed in other similar observational studies of bipolar disorder (Baldessarini et al. 2012; Leboyer et al. 2005; Geoffroy et al. 2013). An index of clinical morbidity was calculated by dividing the log-transformed lifetime number of mood episodes by the log-transformed years of illness, to create an indicator of illness severity. Further, as explained in a previous work, a composite measure of social outcome was computed, weighting social variables as follows: being employed (10 points), currently being married (5 points), and having completed high school (1 point) (Baldessarini et al. 2012).

Comorbidities and clinical characteristics were defined in terms of lifetime presence, except in the cases of the history of substance use disorder and history of anxiety disorder in the Argentinian sample. These two variables were defined as the current presence at the time of interview in the Argentinian sample in comparison with the lifetime presence in the sample from the United States.

Statistical analyses were completed using R software version 3.0.1 (R Foundation, Vienna, Austria). Specific comparative analyses included chi-square test comparisons for categorical variables and unpaired *t*-test comparisons of continuous variables. In the case of a non-normally distributed variable, a non-parametric test, namely the Wilcoxon-Mann-Whitney test, was performed. An *F*-test was used to compare the variances of the onset age distributions between the two samples. Multivariate logistic and linear regression modeling were used to assess whether differences in clinical characteristics remained significantly different upon controlling for demographic factors, including gender, education, marital status, occupational status, and clinical factors, including bipolar subtype and onset age. Further, multivariate logistic regression modeling was used to assess the differential rates of psychotropic medication usage by subtype (mood stabilizer, antipsychotic, antidepressant, benzodiazepine/hypnotic, and other medication) across nationalities, controlling for onset age, age at enrollment, illness duration, gender, total social outcome, and diagnostic subtype.

## Results

We studied Argentinian (*n* = 449) and North American (*n* = 503) bipolar I and II patients that visited a variety of psychiatric hospitals throughout Argentina or who were referred to the Stanford Bipolar Disorders Clinic in suburban Northern California. The Argentinian study participants were 65.0% female, with a mean age of 45.1 ± 13.0 years, and 32.6% were unemployed. The study participants from the United States were 58.3% female, with a mean age of 35.6 ± 13.3 years, and 39.8% were unemployed (Table 1). Accordingly, total social outcome was found to be better in the Argentinian sample as compared to the North American sample (9.3 ± 3.2 vs. 8.2 ± 5.9, *p* = 0.0001).

**Table 1:** **Demographics and clinical characteristics of bipolar disorder patients in Argentina and the United States**

	**Argentina (** ***n*** **= 449)**	**United States (** ***n*** **= 503)**	**Total (** ***n*** **= 952)**
Demographics			
Age at Intake (years) [mean ± SD]	45.1 ± 13.0****	35.6 ± 13.3	40.0 ± 13.2
Females [%,(*n*)]	65.0 (292)*	58.3 (293)	61.4 (585)
Single [%,(*n*)]	61.1 (274)****	36.1 (182)	47.9 (456)
Unemployed [%,(*n*)]	32.6 (146)*	39.8 (200)	36.3 (346)
Less than 8 years of education [%,(*n*)]	19.1 (86)****	1.4 (7)	9.8 (93)
Social Outcome [mean ± SD]	9.3 ± 3.2****	8.2 ± 5.9	8.7 ± 4.8
Clinical characteristics			
Bipolar I [%,(*n*)]	54.9 (247)*	48.3 (243)	51.5 (490)
Early onset (≤21 years) [%,(*n*)]	38.6 (173)****	78.3 (394)	59.6 (567)
Prior year rapid cycling [%,(*n*)]	12.4 (56)****	36.2 (182)	25.0 (238)
Prior suicide attempt [%,(*n*)]	45.7 (205)****	30.8 (155)	37.8 (360)
Prior psychiatric hospitalization [%,(*n*)]	55.0 (247)****	37.8 (190)	45.9 (437)
Substance use disorder [%,(*n*)]^d^	11.0 (49)****	44.5 (224)	28.7 (273)
Anxiety disorder [%,(*n*)]^a^	10.7 (48)****	64.8 (326)	39.3 (374)
Age at onset (years) [mean ± SD]	27.1 ± 11.4****	17.9 ± 8.4	22.3 ± 10.8
Illness duration (years) [mean ± SD]	17.8 ± 11.8	17.6 ± 8.4	17.7 ± 10.2
Morbidity index	0.78***	0.87	0.83
Number of psychotropic medications [mean ± SD]	2.5 ± 1.0	2.6 ± 1.7	2.6 ± 1.4

^a^Discrepancy in measures, as described in ‘Methods’ section. *p < 0.05; **p < 0.01; ***p < 0.001; ****p < 0.0001 versus United States.

**Figure 1:**
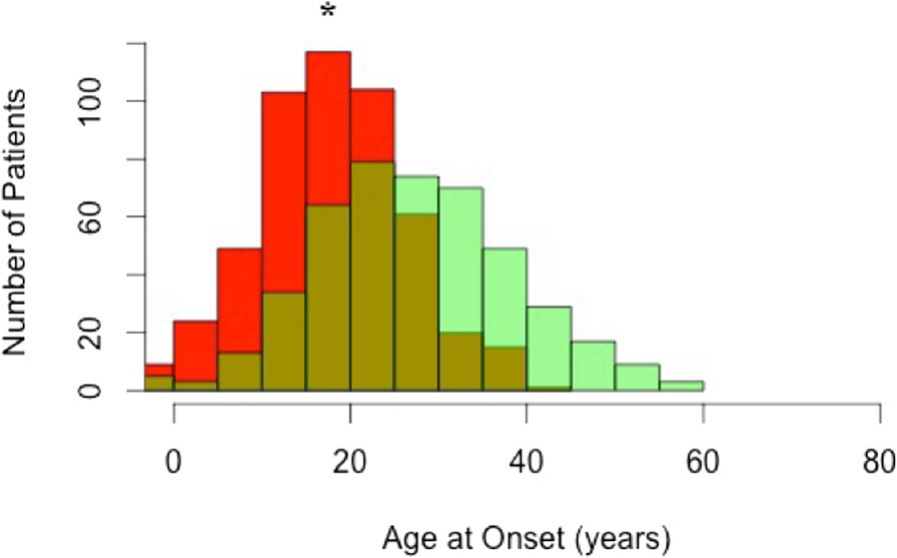
Histogram of bipolar disorder onset ages in Argentina and the United States. Green indicates onset ages in Argentina. Red indicates onset ages in the United States. *p < 0.0001 for difference in means and variances of onset age distributions for Argentina versus United States.

In terms of clinical characteristics and comorbidities, the two samples differed significantly, as well. Patients from Argentina compared to the United States had a higher rate of having bipolar I disorder (54.9% vs. 48.3%, *p* = 0.04). In the North American population, as demonstrated by the almost 10 years earlier mean onset age (17.9 ± 8.4 vs. 27.1 ± 11.4 years, *p* < 0.0001), a significantly greater proportion of patients had an onset age of less than or equal to 21 years of age (78.3% vs. 36.6%, *p* < 0.0001). Accordingly, a greater percentage of the Argentinian sample presented with late onset (>40 years) than the North American sample (14.3% vs. 2.6%, *p* < 0.0001). Further, the variances of the onset age distributions were significantly different between the two samples (*F* = 1.7, *p* < 0.0001) (Figure 1). The sample from the United States also had significantly higher rates of comorbid anxiety disorder (64.8% vs. 10.7, *p* < 0.0001), comorbid substance use disorder (44.5% vs. 11.0%, *p* < 0.0001), and prior year rapid cycling (36.2% vs. 12.4%, *p* < 0.0001), as well as a greater morbidity (*t* = 3.8, *df* = 889, *p* = 0.0002). Conversely, the sample from Argentina had higher rates of prior suicide attempt (45.7% vs. 30.8%, *p* < 0.0001) and history of psychiatric hospitalization (55.0% vs. 37.8%, *p* < 0.0001). However, as noted above, no significant difference was found between Argentina and the United States in terms of the mean illness duration (17.8 ± 11.8 vs. 17.6 ± 8.4 years, *p* = 0.83) or the mean number of psychotropic medications (2.5 ± 1.0 vs. 2.6 ± 1.7, *p* = 0.57). Thus, significant differences at an alpha level of 0.05 were found between the samples for all of the demographics and illness characteristics in Table 1, except for mean illness duration and mean number of psychotropic medications. Using a Bonferroni-corrected *p*-value of 0.003, most of the abovementioned demographic and clinical differences remained significant.

In a comparison of the early-onset subsets (≤21 years of age), the Argentinian sample again showed better social outcomes (9.0 ± 3.5 vs. 7.9 ± 5.9, *p* = 0.03) and higher rates of prior psychiatric hospitalization (57.5% vs. 34.9%, *p* < 0.0001), bipolar I disorder (56.4% vs. 44.3%, *p* = 0.01), and prior suicide attempt (54.7% vs. 34.3%, *p* < 0.0001) (Table 2). Conversely, the sample from the United States again showed higher levels of prior year rapid cycling (40.5% vs. 20.5%, *p* = 0.002), substance use disorder (59.8% vs. 14.6%, *p* < 0.0001), anxiety disorder (69.0% vs. 11.0%, *p* < 0.001), and overall clinical morbidity (*t* = 4.0, *df* = 360, *p* < 0.0001) and morbidity index (0.88 vs. 0.75, *t* = 3.8, *df* = 889, *p* < 0.0001). There was no demonstrated difference between the Argentinian and North American samples in terms of the mean number of psychotropic medications (2.6 ± 1.0 vs. 2.6 ± 1.7, *p* = 0.73).

**Table 2:** **Demographics and illness characteristics in early-onset (≤21 years) subsets of bipolar disorder patients in Argentina and the United States**

	**Argentina (** ***n*** **= 172)**	**United States (** ***n*** **= 393)**	***p*** **value for difference**
Demographic characteristics			
Age at intake (years) [mean ± SD]	39.1 ± 13.0	33.3 ± 12.2	<0.0001
Female [%,(*n*)]	66.7 (115)	58.5 (230)	0.30
Single [%,(*n*)]	67.6 (116)	33.7 (132)	<0.0001
Unemployed [%,(*n*)]	25.8 (44)	40.2 (158)	0.001
Less than 8 years of education [%,(*n*)]	19.4 (33)	1.5 (6)	<0.0001
Social outcome [mean ± SD]	9.0 ± 3.5	7.9 ± 5.9	0.03
Clinical characteristics and comorbidities			
Bipolar I [%,(n)]	56.4 (97)	44.3 (174)	0.01
Prior year rapid cycling [%,(*n*)]	20.5 (35)	40.5 (160)	0.002
Prior suicide attempt [%,(*n*)]	54.7 (94)	34.3 (135)	<0.0001
Prior psychiatric hospitalization [%,(*n*)]	57.5 (99)	34.9 (137)	<0.0001
Substance use disorder [%,(*n*)]^a^	14.6 (25)	59.8 (235)	<0.0001
Anxiety disorder [%,(*n*)]^a^	11.0 (19)	69.0 (271)	<0.0001
Illness duration (years) [mean ± SD]	21.9 ± 12.9	18.9 ± 13.3	0.01
Morbidity index	0.75	0.88	<0.0001
Number of psychotropic medications [mean ± SD]	2.6 ± 1.0	2.6 ± 1.7	0.73

^a^Discrepancy in measures, as described in ‘Methods’ section.

**Figure 2:**
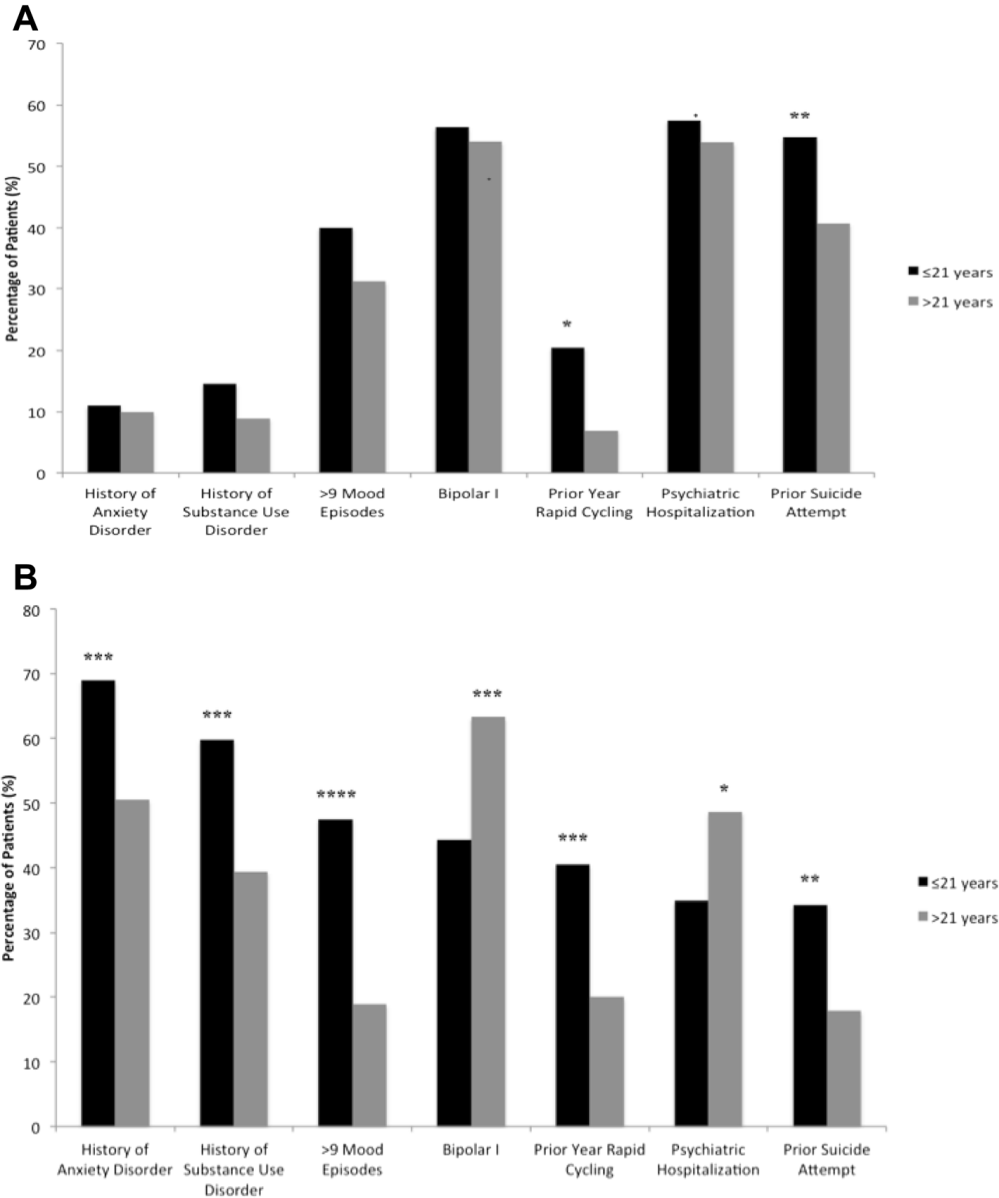
Illness characteristics in early- (≤21 years) compared to later-onset patients in Argentina and the United States. **(A)** Illness characteristics by onset age among patients in Argentina. **(B)** Illness characteristics by onset age among patients in the United States. *p < 0.05; **p < 0.01; ***p < 0.001; ****p < 0.0001 for onset age ≤21 versus >21 years.

**Figure 3:**
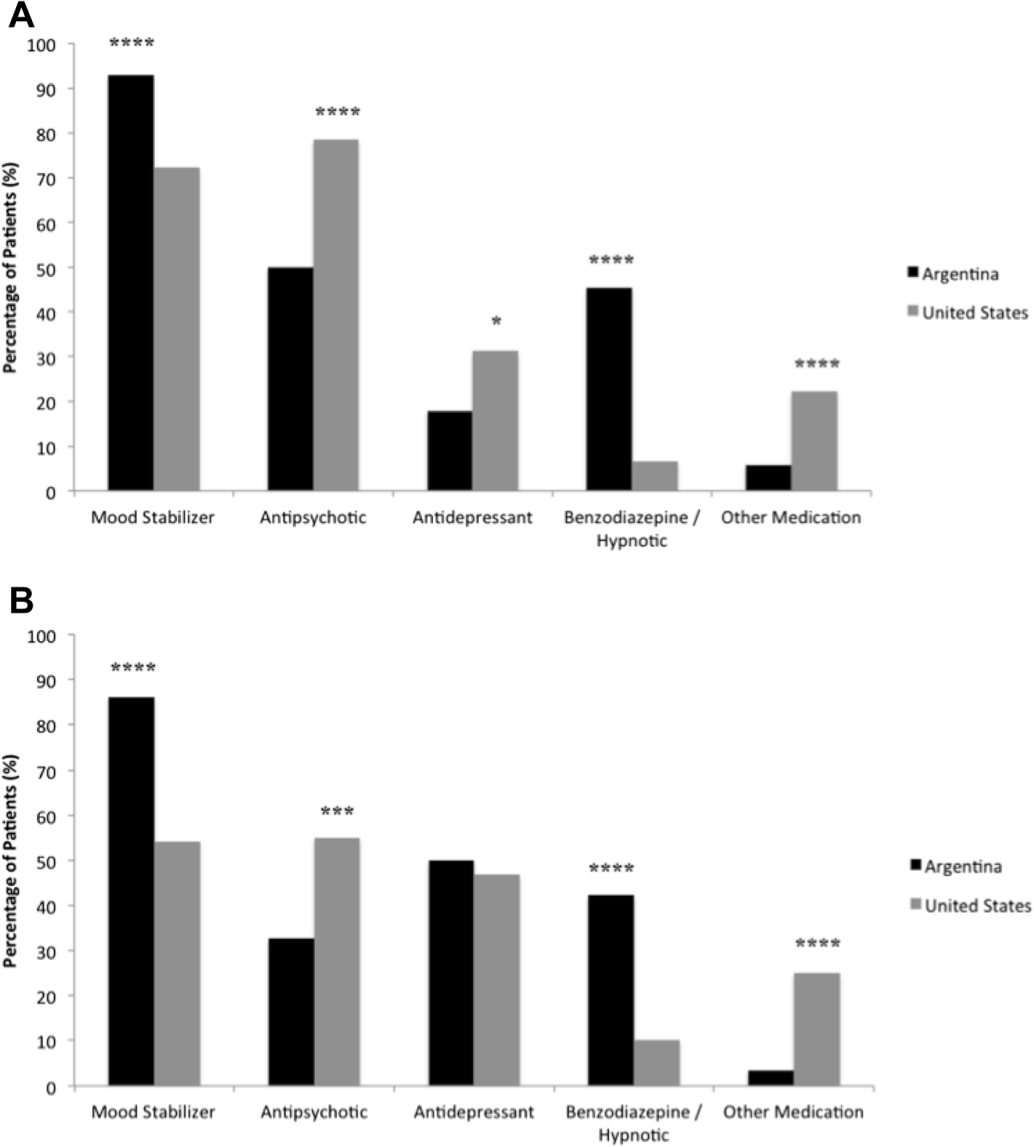
Medication patterns by nationality and bipolar subtype. **(A)** Comparative medication patterns in bipolar I disorder. **(B)** Comparative medication patterns in bipolar II disorder. *p < 0.05; **p < 0.01; ***p < 0.001; ****p < 0.0001 for Argentina versus United States.

Finally, the patterns of results differed substantively in a comparison of unfavorable illness characteristics between early (age ≤21 years) and later onset within the Argentinian and North American samples (Figures 2 and 3). Within the Argentinian sample, the early-onset cohort was only shown to have significantly higher rates of prior year rapid cycling (20.5% vs. 6.9%, *p* = 0.01) and prior suicide attempt (54.7% vs. 40.7%, *p* = 0.005). For all of the other observed variables, the early-onset cohort demonstrated only non-significantly greater rates of unfavorable illness characteristics. In contrast, within the North American sample, significant differences in the rates of unfavorable illness characteristics were demonstrated for all of the observed variables. In all characteristics except bipolar I subtype (44.3% vs. 63.3%, *p* = 0.0007) and prior psychiatric hospitalization (34.9% vs. 48.6%, *p* = 0.01), the early-onset cohort demonstrated a more severe phenotype, including history of anxiety disorder (69.0% vs. 50.5%, *p* = 0.0005), history of substance use disorder (59.8% vs. 39.4%, *p* = 0.0002), history of more than nine lifetime mood episodes (47.5% vs. 18.9%, *p* < 0.0001), prior year rapid cycling (40.5% vs. 20.0%, *p* = 0.0002), and prior suicide attempt (34.3% vs. 17.9%, *p* = 0.002).

Prescription patterns between the United States and Argentina were computed in aggregate as well as when stratifying by illness subtype (bipolar I vs. bipolar II) and by illness onset age (≤21 vs. >21 years). Independent of stratification by potential confounding demographic and illness characteristics, the Argentinian compared to the American sample was found to be taking significantly more mood stabilizers and benzodiazepines/hypnotics, and significantly fewer antipsychotics and other psychotropic medications. No significant differences in terms of antidepressant use were found between the two nationalities when considered in aggregate. Analysis of prescription patterns between Argentina and the United States explored within subdivisions of bipolar subtype indicated that among patients with bipolar I disorder, rates of prescription of all five medication subgroups differed significantly (Figure 3a), whereas among patients with bipolar II disorder, rates of prescription of all medication classes except antidepressants differed significantly (Figure 3b). The consistency of the abovementioned prescribing patterns was further confirmed using a multivariate logistic regression model, controlling for onset age, age of enrollment, illness duration, gender, total social outcome, and diagnostic subtype. Across both illness subtypes, Argentinian compared to North American patients had significantly higher rates of taking mood stabilizers and benzodiazepines/hypnotics and significantly lower rates of taking antipsychotics and other medications.

Upon performing multivariate logistic regression analyses controlling for demographic characteristics as well as bipolar subtype, all differences in clinical characteristics between the populations were found to persist.

## Discussion

Overall, the two samples of bipolar patients derived from Argentina and the United States differed significantly not only in terms of demographic characteristics but also in terms of clinical features. Despite the similar sample sizes, the Argentinian sample had many more unfavorable demographic characteristics, including lower marriage rates and less years of education. It is worth noting that almost 20% of individuals had not completed primary education. Such low levels of education may warrant a more careful examination of the effects of bipolar disorder on the attainment and sustainment educational opportunities, as well as further development of programs to support social engagement for individuals with severe mental illness. However, when a cumulative measure of social outcome was computed, the Argentinian sample demonstrated a more favorable social outcome. A recent study found that married women with bipolar disorder had lower cumulative illness severity and fewer prior mood episodes than never-married women with bipolar disorder, suggesting that patients may be sensitive to the beneficial effects of social support (Lieberman et al. 2010). As such, promoting a positive social environment for individuals with bipolar may support positive therapeutic outcomes.

In addition to the demographic characteristics, the two populations also differed significantly with regard to clinical characteristics. In terms of bipolar disorder onset age, almost 80% of the North American population presented with onset at less than or equal to 21 years of age, in contrast in the Argentinian population, only 40% of patients presented with such an early onset. This relevant difference in distribution of onset age as a categorical parameter was consistent with the nearly 10-year difference in mean onset age as a continuous parameter between the two samples. Also consistent with the difference in mean onset age, onset in later adulthood, defined as after 40 years of age, was substantially more prevalent in the Argentinian sample. Some investigators have proposed that a late-onset phenotype of bipolar disorder may represent a different etiological pathway, favoring organic brain disease over family history of mood disorder (Almeida and Fenner 2002). However, proponents of this theory noted that few clinical differences appear between the earlier- and later-onset groups, and that those present are potentially due to differences in illness duration (Baldessarini et al. 2012).

The Stanford and Argentinian cohorts had complex differences with respect to unfavorable illness characteristics. The lower onset age seen in the Stanford compared to Argentinian cohort has been associated with multiple unfavorable illness characteristics. Indeed, the Stanford cohort compared to Argentinian cohort had a significantly higher morbidity index and significantly higher rates of prior year rapid cycling, substance use disorder, and anxiety disorder (although methodological differences could have also contributed to the latter two comorbidity differences). Although the significantly lower rate of bipolar I disorder could account for the Stanford compared to Argentinian cohort significantly less often having a history of prior psychiatric hospitalization, the potential factors contributing to the Stanford compared to Argentinian cohort having a significantly lower rate of prior suicide attempt remains to be established.

It is possible that the significant demographic and bipolar subtype differences between the two samples contributed substantively to the observed clinical differences. For instance, bipolar I disorder and psychiatric hospitalization, as well as bipolar II disorder and rapid cycling, are commonly highly correlated, and as such, the different rates of bipolar subtypes between the samples could have contributed substantively to these observed differences. The higher rate of prior year rapid cycling in the Stanford compared to Argentinian cohort (36.2% vs. 12.4%) could be related to the higher rate of early onset (≤21 years) in the Stanford compared to Argentinian cohort (78.3% vs. 38.6%). However, the difference in rates of prior year rapid cycling did not appear to be related to rate of antidepressant administration, which was similar across the two cohorts.

Nevertheless, upon performing multivariate logistic regression analyses controlling for demographic characteristics as well as bipolar subtype, all differences in clinical characteristics between the populations were found to persist. The results of these regression analyses suggest that the observed differences in clinical characteristics, including rates of prior psychiatric hospitalization, prior year rapid cycling, prior suicide attempt, anxiety disorder, substance use disorder, and current medication utilization were not simply a reflection of differences in demographic characteristics and bipolar subtype between the national samples.

In our study, despite the significant differences in demographic and clinical characteristics (including onset age), there was no difference in illness duration between the samples from Argentina and the United States. This finding supports a greater role of onset age than illness duration in determining the severity of the illness course. However, substantial controversy remains as to the respective roles of onset age and illness duration in the determination of prognosis among bipolar disorder patients. Some studies seeking to identify clinical stages in bipolar disorder by determining combinations of predictive factors have found that there was no difference in mean illness duration between groups identified with ‘good’ versus ‘poor’ outcomes (Reinares et al. 2013). However, other studies have found illness duration to be inversely correlated with the likelihood and speed of recovery (Peters et al. 2014) as well as with gray matter volume, suggesting a potential neurodegenerative progression of bipolar disorder across the clinical course (Frey et al. 2008). Though the role of illness duration in the determination of potential neuroprogression has not yet been definitively identified, our findings support onset age as a more significant predictor of poor illness course than total illness duration.

An exploration of the rates of current usage of different medication subtypes revealed interesting, highly significant differences in the patterns of prescription between the two samples. Even when controlling for potential clinical and demographic differences between the two samples that could contribute substantively to observed differences in medication patterns, Argentinians compared to North Americans were more often taking mood stabilizers and benzodiazepines/hypnotics and less often taking antipsychotics. Such differences may reflect significant trans-national variations in the prescription of different types of medications for the treatment of the same disorder.

Given documented differences in treatment patterns by bipolar subtype, we were interested to further explore whether differences in medication patterns appeared within bipolar I and bipolar II subgroups (Ghaemi et al. 2006). This analysis revealed that the significant differences between national patterns of medication indeed persisted upon dividing the Argentinian and North American samples by bipolar subtype. As such, it can be suggested that the observed transnational differences in rates of prescription of the medication subgroups reflected actual differences in treatment patterns as opposed to differences in the distribution of bipolar subtype. Further exploration of the therapeutic implications of different treatment regimens is warranted. Investigating differential rates of recurrence between the populations as a measure of functional outcome with different treatment regimens could provide interesting insights into the comparative effectiveness of various medication subtypes in the treatment of bipolar disorder.

In addition to the comparison between the entire Argentinian and North American samples, we performed a more specific comparison between only the subgroups with early onset, defined as occurring at less than or equal to 21 years of age. Early onset has consistently been associated with a worse illness course (Leverich et al. 2007); as such, comparing the two groups with the worst prognosis was thought to have the potential to reveal notable differences between the populations. A majority of the same demographic differences observed for the entire groups persisted between the early-onset subgroups, though in some instances to a lesser degree of significance. Even when controlling for differences in demographic characteristics and bipolar subtype, the Argentinian patients with early onset were found to have been previously hospitalized for psychiatric treatment and have prior suicide attempt more often. In contrast, the North American individuals presented with more severe clinical morbidity, higher rates of prior year rapid cycling, history of substance use disorders, and history of anxiety disorder. As such, individuals with early onset in neither Argentina nor the United States presented consistently with a worse illness course; each group presented with more and less severe clinical characteristics in differing aspects.

In performing comparisons between sites, particularly between different countries, it must be noted that not every clinical characteristic was measured in a consistent manner. In this study, there was a discrepancy in the measurement of rates of comorbid anxiety and substance use disorders. Though both groups used the same diagnostic criteria, the Argentinian site defined this variable as the current presence of anxiety or substance abuse disorders at the time of interview, while the North American site used a definition of lifetime presence of an anxiety or substance use disorder. Therefore, though the direct comparison of these rates between nationalities may have validity limitations, the overall rate of comorbidity within each sample and the comparison within each nationality between onset-age groups may still be of interest. Specifically, it is worth noting that almost 45% of the patients from the United States suffered at one point in their lives from a comorbid substance use disorder. Further, almost 65% of the North American cohort had a comorbid anxiety disorder prior to enrollment in the study. More investigation is warranted to analyze the rates of these comorbidities within subgroups of bipolar patients so as to assess the potential inclusion of these characteristics in diagnostic criteria (Vazquez et al. 2014).

Lastly, a comparison was performed, within each national group, between individuals with early (≤21 years) versus later (>21 years) onset of bipolar disorder to determine whether onset age was a reliable predictor of unfavorable illness characteristics in both Argentina and the United States. In the North American sample, early onset was more robustly associated with the presence of comorbidities and unfavorable illness characteristics compared to the later-onset group. The early-onset subset presented significantly higher rates of comorbid anxiety disorder, substance abuse disorder, prior year rapid cycling, and prior suicide attempt, as well as a significantly greater portion of the sample with at least ten lifetime mood episodes. In contrast, the later-onset group had higher rates of bipolar I and of psychiatric hospitalization. Previous findings have varied as to the relationship between bipolar subtype and age at onset, reflecting varying differences in age at illness presentation (Benazzi 1999). Onset age within the North American sample therefore consistently differentiated distinct illness profiles.

However, within the Argentinian sample, such a broad pattern of significant differences in the prevalence unfavorable illness characteristics was not revealed. The early-onset group showed significantly elevated rates of only prior year rapid cycling and prior suicide attempt. Importantly, these onset-age-related differences persisted between the groups when controlling for bipolar subtype, suggesting that these differences were not simply a reflection of the distinctive clinical presentation of the two bipolar subtypes. The other traits that were examined did not reveal significant differences in the rate of occurrence between early- and later-onset groups, though the early-onset group did show non-significantly elevated rates of each characteristic. It is possible that these differences were non-significant due to a lack of statistical power, given that only 172 Argentinian patients presented with early onset in comparison to 393 North American patients, though this is an unlikely explanation as seen by the highly similar rates between onset-age groups in the Argentinian sample. Given the apparent difference in the relevance of onset age as a predictor of distinct illness course, it is necessary to consider individual sample characteristics before utilizing onset age as a predictive variable. Despite the differential relevance of onset age in predicating the severity of comorbidities between the two samples, onset age appears to be a relatively robust variable for identifying a more severe illness course.

This study possesses numerous strengths, including a substantial number of patients, use of validated assessment instruments, and use of largely consistent measures across sites. Further, when the significance of results was re-computed using a Bonferroni correction for multiple comparisons, most of the observed differences remained significant. However, our analysis also had noteworthy limitations, several of which are typical of observational and comparative studies. First, selection bias may have been introduced into the study due to the significant demographic differences between the two sites, although the impacts of such differences were limited by use of regression analyses that covaried for demographic differences. The Bipolar Disorder Clinic at Stanford University is a tertiary-care private clinic in suburban Northern California. The patient population has relatively high socioeconomic and educational levels. In contrast, the patients from Argentina were enrolled at both public and private psychiatric hospitals and clinics that generally had lower socioeconomic and educational levels. Therefore, our cross-national comparison findings may be limited by socioeconomic differences, although the persistence of clinical differences in the analyses that covaried for demographic and illness subtype differences make this potential limitation less problematic. Despite the demographic differences, it is of note that both the South and North American samples consisted solely of outpatients.

Further, the results of this study should be interpreted carefully, noting that such findings cannot be assumed to uniformly represent the state of the entire countries of Argentina or the United States. Not only did the two samples differ from one another but they are also not reflective of the overall demographic makeup of the two countries either. As such, the differences between the samples are presented as a means to highlight the variations in clinical presentation between the two study samples but not necessarily as a means to represent the residents of a country as a whole.

Additionally, as with most studies of this kind, the definition of onset age was determined by retrospective recall rather than in a prospective manner. Therefore, a potential degree of uncertainty may have been introduced into the samples due to the effects of recall bias, though it is important to note that onset age in both studies was determined in the same manner. The determination of age at onset may also be affected in part by the extent of delay to diagnosis following onset of mood symptoms, which may differ by region of the world (Wang et al. 2007) but was not measured in either of the samples; for this reason, onset age was defined as the first recalled occurrence of syndromic mood dysregulation, rather than the age at diagnosis or initiation of treatment. Similarly, the validity of retrospectively recalled number of lifetime mood episodes was augmented by assessing it as a categorical variable (<9 lifetime mood episodes), rather than as a continuous variable.

## Conclusions

The bipolar samples from Argentina and the United States differed significantly with respect to several demographic and clinical characteristics. Broadly, the Argentinian sample tended to have more unfavorable demographic characteristics, while the North American sample presented with in several substantive ways more unfavorable illness characteristics (in spite having more favorable demographic characteristics). Despite these specific differences, both samples presented important and distinctive challenges for diagnosis and treatment. In order to address these challenges, onset age may be a valid and robust measure for identifying a more severe illness course, though the specific characteristics of each sample must be taken into account. Specifically, onset in youth may be earlier and may have more robust associations with unfavorable illness characteristics in North compared to South America. Further study is warranted to determine the extent to which onset age should be used in the international context as a defining characteristic for more homogenous subgroups within bipolar disorder.
